# Study protocol of the TIRED study: a randomised controlled trial comparing either graded exercise therapy for severe fatigue or cognitive behaviour therapy with usual care in patients with incurable cancer

**DOI:** 10.1186/s12885-017-3076-0

**Published:** 2017-01-28

**Authors:** Hanneke Poort, Constans A. H. H. V. M. Verhagen, Marlies E. W. J. Peters, Martine M. Goedendorp, A. Rogier T. Donders, Maria T. E. Hopman, Maria W. G. Nijhuis-van der Sanden, Thea Berends, Gijs Bleijenberg, Hans Knoop

**Affiliations:** 1 Expert Center for Chronic Fatigue, Radboud university medical center, Nijmegen, The Netherlands; 2 Department of Medical Oncology, Radboud university medical center, Nijmegen, The Netherlands; 3 Department of Health Psychology, University Medical Center Groningen, University of Groningen, Groningen, The Netherlands; 4 Department of Health Evidence, Radboud university medical center, Nijmegen, The Netherlands; 5 Department of Physiology, Radboud university medical center, Nijmegen, The Netherlands; 6 Institute for Health Sciences, IQ Healthcare, Radboud university medical center, Nijmegen, The Netherlands; 7 Department of Medical Psychology, Amsterdam Public Health research institute, Academic Medical Centre (AMC), University of Amsterdam, Amsterdam, The Netherlands; 8 Expert Center for Chronic Fatigue, Department of Medical Psychology, Amsterdam Public Health research institute, VU University Medical Center, Amsterdam, The Netherlands

**Keywords:** Fatigue, Advanced cancer, Graded exercise therapy, Cognitive behaviour therapy, Randomised controlled trial

## Abstract

**Background:**

Fatigue is a common and debilitating symptom for patients with incurable cancer receiving systemic treatment with palliative intent. There is evidence that non-pharmacological interventions such as graded exercise therapy (GET) or cognitive behaviour therapy (CBT) reduce cancer-related fatigue in disease-free cancer patients and in patients receiving treatment with curative intent. These interventions may also result in a reduction of fatigue in patients receiving treatment with palliative intent, by improving physical fitness (GET) or changing fatigue-related cognitions and behaviour (CBT). The primary aim of our study is to assess the efficacy of GET or CBT compared to usual care (UC) in reducing fatigue in patients with incurable cancer.

**Methods:**

The TIRED study is a multicentre three-armed randomised controlled trial (RCT) for incurable cancer patients receiving systemic treatment with palliative intent. Participants will be randomised to GET, CBT, or UC. In addition to UC, the GET group will participate in a 12-week supervised exercise programme. The CBT group will receive a 12-week CBT intervention in addition to UC. Primary and secondary outcome measures will be assessed at baseline, post-intervention (14 weeks), and at follow-up assessments (18 and 26 weeks post-randomisation). The primary outcome measure is fatigue severity (Checklist Individual Strength *subscale fatigue severity*). Secondary outcome measures are fatigue (EORTC-QLQ-C30 *subscale fatigue*), functional impairments (Sickness Impact Profile *total score*, EORTC-QLQ-C30 *subscale emotional functioning, subscale physical functioning*) and quality of life (EORTC-QLQ-C30 *subscale QoL*). Outcomes at 14 weeks (primary endpoint) of either treatment arm will be compared to those of UC participants. In addition, outcomes at 18 and 26 weeks (follow-up assessments) of either treatment arm will be compared to those of UC participants.

**Discussion:**

To our knowledge, the TIRED study is the first RCT investigating the efficacy of GET and CBT on reducing fatigue during treatment with palliative intent in incurable cancer patients. The results of this study will provide information about the possibility and efficacy of GET and CBT for severely fatigued incurable cancer patients.

**Trial registration:**

NTR3812; date of registration: 23/01/2013.

## Background

Cancer is a leading cause of mortality worldwide, with 8.2 million deaths in 2013 [1]. As a result of improvements in treatment options for certain cancers, substantial progress has been made in curative treatment of cancer. Despite these positive developments, a substantial subgroup of cancer patients will (eventually) be diagnosed with incurable cancer. The medical treatment of incurable cancer has a palliative intention, with prolonging life as one of its main aims [2]. For some cancer types, advances in cancer treatment with palliative intent have resulted in an extended period of life, resulting in more long-term or chronic cancer treatment. Next to prolonging life, treatment of incurable cancer should also be aimed at maintaining quality of life for as long as possible and relieving physical and psychological symptoms [2]. As a result of the longer-term treatment of incurable cancer patients, aspects regarding quality of life and symptom management become even more important.

### Fatigue in patients with incurable cancer

Fatigue is one of the most commonly reported symptoms during systemic treatment for incurable cancer, being reported by up to 99% of patients [3–7]. There are various ways to define fatigue, but cancer-related fatigue (CRF) is a term that is most widely used to address this symptom. The National Comprehensive Cancer Network (NCCN) defines CRF as *“a distressing, persistent, subjective sense of physical, emotional and/or cognitive tiredness or exhaustion related to cancer or cancer treatment that is not proportional to recent activity and interferes with usual functioning” *[8]. Studies show that CRF is among the most distressing symptoms [3, 9, 10] and is associated with reduced quality of life, poor performance status, and difficulty performing daily activities [3, 4, 11]. Many factors are likely to contribute to CRF in patients with incurable cancer. The multiple causes of CRF can result from the underlying disease, from secondary factors such as anaemia, infection, dehydration, and treatment side effects, or from loss of muscle mass. Apart from these physical factors, depression and anxiety can also contribute to CRF. There is also evidence suggesting that cognitive and behavioural factors, such as sleeping problems, fatigue catastrophising, and inappropriate coping are related to fatigue in patients with incurable cancer [12].

Management of CRF in incurable cancer patients should first focus on identifying and treating somatic causes, for example anaemia or hypothyroidism [8]. Yet, often no somatic cause for CRF can be found. When no somatic cause can be identified, the management of CRF can involve pharmacological treatment or non-pharmacological interventions. Thus far, no recommendation for a specific drug treatment for fatigue in palliative care patients could be given [13]. There is also no evidence-based non-pharmacological intervention for CRF in incurable cancer patients. Two non-pharmacological approaches, Graded Exercise Therapy (GET) and Cognitive Behaviour Therapy (CBT), seem promising interventions based on findings from studies addressing CRF in other cancer patients that will be discussed below.

### Exercise interventions for CRF in cancer patients

In contrast to the old advice to ‘get plenty of rest’ during cancer treatment, patients are now encouraged to optimise levels of physical activity [8]. A low level of physical activity during cancer treatment can lead to decreased physical functioning by a substantial loss of cardiopulmonary fitness and muscle mass [14]. On the other hand, increasing physical activity has been suggested as helpful in reducing CRF by improving physical capacity, resulting in a reduced effort to perform everyday activities [8]. Cramp & Byron-Daniel (2012) suggested that exercise interventions can help to reduce CRF both during and after adjuvant treatment for cancer [15]. Efficacy of exercise interventions for the subgroup of patients receiving cancer treatment with palliative intent was not examined in this Cochrane systematic review. Nonetheless, a systematic review by Lowe et al. (2009) did provide evidence that exercise interventions are feasible in patients with incurable cancer as the majority of participants were able to tolerate various physical activity interventions [16]. Three of the six reviewed studies had fatigue as one of the outcome measures and all three reported a reduction in fatigue [17–19]. However, the methodological quality of these pilot studies was evaluated as poor and only one study had a control condition [16].

Following the NCCN recommendations for exercise programs, our research group developed a 6-week GET intervention that was tailored to the physical fitness level of each participant and began at a low level of intensity and duration, progressed slowly, and was modified when the participant’s condition changed. This intervention was tested for feasibility and efficacy was explored in an uncontrolled pilot study of 26 incurable cancer patients. GET was not only feasible in terms of participants’ adherence and evaluation, but also efficacious with significant improvements in self-reported fatigue and quality of life [20]. A large-scale randomised controlled trial (RCT) is needed to confirm these promising results.

### Cognitive behaviour therapy for CRF in cancer patients

Most research on the efficacy of CBT for CRF has been done in cancer survivors or cancer patients receiving cancer treatment with curative intent. Systematic reviews and meta-analyses have indicated that CBT can reduce fatigue in cancer survivors [21, 22]. Two RCTs performed by our research group have demonstrated that fatigue and functional impairments in severely fatigued cancer survivors can be significantly reduced by CBT for CRF [23, 24]. This fatigue-specific intervention targets several cognitive-behavioural perpetuating factors of CRF. The intervention is based on the underlying assumption that cancer treatment and/or the cancer itself may trigger fatigue (precipitating factors), but that other factors such as sleep disturbance, physical inactivity, and dysfunctional thoughts about fatigue might be responsible for the persistence of fatigue (perpetuating factors) [25]. Positive intervention effects of CBT for CRF were sustained at 2-years follow-up [26]. The efficacy of CBT for CRF compared to usual care was also assessed in an RCT aimed at cancer patients during cancer treatment with curative intent [27]. Despite a significant reduction in fatigue immediately after the intervention for patients in the CBT arm, no differences were observed between these two conditions at follow-up with effects diminishing after seven months [28]. It should be noted though, that being severely fatigued was not an entry criterion for this RCT, and thus a floor effect may be present in this trial.

While there are no RCTs to date that investigated the efficacy of CBT specifically aimed at reducing fatigue in incurable cancer patients receiving cancer treatment with palliative intent, two previous RCTs provide indirect support for the positive effects of CBT on fatigue outcomes in a sample of cancer patients of whom a subgroup had incurable cancer [29, 30]. Although these RCTs did show an overall effect on fatigue, it is not clear whether this can be generalised to the group of cancer patients receiving treatment with palliative intent since subgroup analyses were not performed. Based on our previous experience with CBT for CRF in both cancer survivors and patients receiving cancer treatment with curative intent, and results of a recent study which suggested that the same perpetuating psychosocial factors are associated with fatigue in patients receiving cancer treatment with palliative intent [12], we think it is important to examine the efficacy of CBT for CRF in an RCT for this new target population.

### The role of physical activity and fitness versus fatigue-related cognitions as mediators of the reduction in CRF

Exercise interventions aiming to reduce CRF in cancer patients are based on the assumption that a lack of physical activity and deconditioning during cancer treatment can worsen fatigue [31]. It is assumed that with exercise interventions physical activity and fitness can be increased, resulting in a reduction in CRF. CBT aimed at reducing CRF in cancer patients is based on the assumption that several fatigue-related cognitions (i.e., low self-efficacy and catastrophising thoughts) and behaviours are related to the persistence of fatigue [25]. Targeting cognitions with CBT is assumed to result in less dysfunctional thoughts about fatigue, which contributes to the reduction in CRF. Although these assumptions are widespread, the role of an increase in physical activity and fitness versus a change in fatigue-related cognitions in reducing CRF has not yet been investigated in interventions for patients with incurable cancer. To investigate which factors contribute to a reduction in CRF, mediation analysis can be helpful. This technique provides insight into which factors mediate the expected reduction in CRF brought on by GET on CRF. Mediation analysis can thereby help us to better understand how interventions work [32].

### Aims of the TIRED study

We designed a multicentre RCT to test the efficacy of either GET or CBT compared to Usual Care (UC) in reducing fatigue (primary outcome) in incurable cancer patients receiving systemic treatment with palliative intent. In addition, the efficacy on improving quality of life and functional impairment will be studied. All outcomes will be assessed at baseline, and at 14, 18 and 26-weeks post-randomisation. We will assess the efficacy of GET or CBT compared to UC directly post-intervention at 14-weeks post-randomisation, which is the primary endpoint of this study. In addition, we will determine whether the expected intervention effects are sustained at follow-up assessments (18-weeks and 26-weeks post-randomisation). Furthermore, if GET and/or CBT are efficacious in reducing CRF, we will perform a mediation analysis to test if the changes in four variables (i.e., physical activity, physical fitness, self-efficacy with respect to fatigue, and/or fatigue catastrophising) mediate the reduction in fatigue.

## Methods

### Design

A non-blinded multicentre RCT (the TIRED study) will be conducted to evaluate the efficacy of GET and CBT compared to UC for severely fatigued incurable cancer patients receiving cancer treatment with palliative intent.

### Participants

Inclusion and exclusion criteria are listed in Table 1. Patients diagnosed with incurable cancer, receiving systemic treatment with palliative intent, and with a cancer treatment plan based on an expected survival of at least 6 months as judged by their oncologist, will be further assessed for eligibility by nurses and oncologists. We will include patients diagnosed with one of the following cancer types: breast, colorectal, prostate, renal cell, bladder, endometrial, ovarian, cervical, bone and soft tissue, or melanoma. Systemic cancer treatment may include chemotherapy, hormone therapy, targeted therapy, and/or immunotherapy, possibly combined with surgery and/or radiotherapy. The presence of severe fatigue reflected by a score of 35 or higher on the subscale fatigue severity of the Checklist Individual Strength (CIS-fatigue) will be used as a criterion for study entry [33].

**Table 1:** Inclusion and exclusion criteria

Inclusion criteria	
(1)	Age ≥ 18 years.
(2)	Able to read, speak and write the Dutch language.
(3)	Diagnosis of incurable cancer (i.e. breast, colorectal, prostate, renal cell, bladder, endometrial, ovarian, cervical, bone and soft tissue cancer, or melanoma).
(4)	Scheduled for or receiving systemic cancer treatment with palliative intent (i.e., chemotherapy, and/or hormone therapy, and/or targeted therapy, and/or immunotherapy, possibly combined with surgery and/or radiotherapy).
(5)	Cancer treatment plan based on an expected survival of ≥ 6 months as judged by their oncologist.
(6)	Severely fatigued (CIS-fatigue score ≥ 35).
Exclusion criteria	
(1)	Treatable somatic cause that could explain the presence of severe fatigue (other than the underlying disease and the cancer treatment itself).
(2)	Karnofsky Performance Status < 70.
(3)	Symptomatic brain metastases.
(4)	Severe cognitive problems.
(5)	Not able to walk at least 6 min successively.
(6)	Contra-indication for physical exercise.
(7)	Current treatment by a psychiatrist or psychologist for a psychiatric disorder.

### Recruitment

Nurses and oncologists working at oncology outpatient clinics of two University-affiliated hospitals and seven Regional hospitals in the Netherlands will recruit patients. Patients will be screened for the presence of severe fatigue as part of clinical care by administering the CIS-fatigue prior to the start or during systemic treatment with palliative intent when patients visit the outpatient clinic. When eligible patients are severely fatigued, the nurse or oncologist will present the TIRED study by giving patients written information and solicit permission to have a researcher contact them. Those patients who agree to be contacted will be called by the coordinating researcher (HP), who will further inform them about the details and purpose of the study and invite them to participate. A follow-up phone call will be scheduled one week after the first phone call to address questions and determine if patients are willing to participate.

### Procedure

**Fig. 1:**
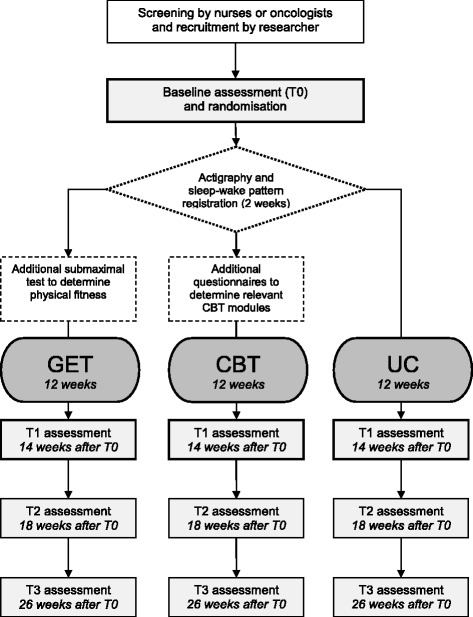
Flowchart of the TIRED study

Eligible patients willing to participate in the study will be asked to sign informed consent upon which they will be invited by a research assistant to complete the baseline assessment (T0) at their own hospital. Upon completion of T0, the research assistant will use a central web-based randomisation service to randomly allocate a participant to one of the three study arms: (1) GET in addition to UC; (2) CBT in addition to UC; or (3) control group receiving UC (see Fig. 1). Participants assigned to GET or CBT will start the intervention approximately two weeks after T0. Both interventions will be delivered at or near their own hospital over a period of 12 weeks. Participants assigned to CBT will complete a set of additional questionnaires to determine relevant intervention modules prior to the first intervention session. Participants assigned to GET will complete an additional submaximal test to determine physical fitness during the first intervention session. At 14 weeks, participants are invited by the research assistant to complete the post-intervention assessment (T1) at the hospital. Follow-up assessments at 18 weeks (T2) and 26 weeks (T3) are entirely web-based and will be completed at home. For participants that do not have Internet access, a paper version of the follow-up questionnaires will be send to their home address, which can be returned in a self-addressed, pre-stamped envelope.

### Randomisation

A central web-based randomisation service provided by an independent statistician will be used. Randomisation will be stratified by centre. We will use block randomisation to reach the same number of participants in all study arms. The ordering of blocks and their respective size will be unknown for the research assistants and coordinating researcher. When possible, minimisation on gender will be performed in order to balance the gender distribution in all study arms. If block randomisation restricts the choice to two or only one study arm, minimisation will always be overruled by block randomisation. A research assistant will perform allocation upon completion of T0 in the presence of the participant.

### Interventions

#### Graded exercise therapy

Participants assigned to the GET group will receive a 12-week supervised exercise programme in addition to UC. The treatment protocol *‘GET for fatigue in incurable cancer patients’* was developed by the study investigators in cooperation with a physiologist (MH) and physical therapist (MN) experienced in exercise programmes for cancer patients. The treatment protocol was based on the protocol for a previous pilot-study in patients with incurable cancer [20]. Physical therapists affiliated with the participating hospitals or from local physical therapy centres will deliver the GET. All therapists will be instructed about the treatment protocol and use of registration forms before enrolment of participants. Throughout the study, supervision will be provided upon request by a physical therapist (MN).

GET will be given by physical therapists individually or in small groups with a maximum of 5 participants, depending on the accrual rate. During the intake session, the physical therapist will collect information about a participant’s physical fitness level (by means of a submaximal test) and physical limitations. Participants will formulate treatment goals in activities of daily living together with the physical therapist, such as performing activities or leisure interests in the foreseeable future that are currently difficult to perform because of a lack of muscle strength or cardiopulmonary fitness. After the intake session, participants will receive weekly two-hour sessions of individually graded training supported by a physical therapist and adjusted to their abilities. In order to adjust the training to an individual participant, their heart rate reserve (HRR) and muscle strength (by means of one-repetition maximum [1RM] tests) will be determined during first session and after every three sessions. The two-hour GET sessions will include a warming up (10 min), high intensity aerobic interval training (35 min), a break (15 min), resistance training (35 min), and a cooling down (10 min). Additionally, there are 15 min available for evaluation of the GET session. In addition to this supervised session, participants are offered to practise in a second weekly session. After every three sessions, training progress will be evaluated and the programme will be adjusted by means of the newly determined HRR and 1RM and discussion of formulated treatment goals.

##### Aerobic training

The aerobic training will consist of cycling on an interval basis prior to the resistance training. Intervals will include alternated bicycling for four minutes at 60% (increasing to 80%) of participants’ HRR with three minutes on 35% (increasing to 50%) of HRR. Heart rate will be monitored during the aerobic training using a Polar® breast band (Polar T31 Breast Band, 2008, Polar Electro, Finland). We will use the Borg Scale of Perceived Exertion after each cycling interval to gauge the perceived intensity of the aerobic training [34].

##### Resistance training

The resistance program will include a circuit of seven exercises targeting large muscle groups important for activities of daily living. The following exercises will be executed: (1) leg press; (2) lunge; (3) vertical row; (4) lateral pull down; (5) abdominal crunch; (6) pull over; and (7) bench press. Exercises will be executed at 60-80% of participants’ 1RM and will consist of 3 sets of 8 to 12 repetitions. Some exercises will be performed more often based on the participants’ difficulties in this area and his or her goals in activities of daily living. Progression will be conducted by the graded activity principle, which states that the focus is on successes and positive experiences and that negative experiences will be prevented as much as possible [35].

#### Cognitive behaviour therapy

Participants in the CBT group will receive *‘CBT for fatigue in incurable cancer patients’* in addition to UC. This intervention was developed by the study investigators based on the evidence-based protocol of CBT for post-cancer fatigue [23, 26]. Adaptations were done for application with our new target population. This adapted CBT will consist of a maximum of ten sessions over a period of 12 weeks (i.e., one assessment session and maximum nine individual one-hour face-to-face treatment sessions). Qualified and trained psychologists will deliver CBT for fatigue. Prior to intervention delivery, all therapists will receive a three-day training provided by two experienced clinical psychologists (HK and TB). This training will provide background and rationale for each of the intervention modules and involves role-playing to practise the intervention components. An experienced clinical psychologist (HK) will provide on-going supervision to CBT therapists throughout the study.

CBT for fatigue in incurable cancer patients includes several modules aimed at fatigue-perpetuating cognitions and behaviours. Participants randomised to CBT will complete a set of additional questionnaires prior to the first intervention session to assess potential perpetuating factors (see Table 2). During the first intervention session it will be determined by the therapist which factors are applicable for the particular patient, which leads to a tailored-made intervention as only the relevant treatment modules will be selected. The goal of CBT is reduction of severe fatigue and fatigue-related disability. All participants will start with setting their treatment goals. Participants will be helped to formulate concrete goals in behavioural terms, such as resuming activities or leisure interests in the foreseeable future that are discontinued because of being severely fatigued. Then, therapists and participants will work on adjusting the fatigue-perpetuating factors that are applicable to the individual participant: (1) sleep problems and deregulated sleep-wake cycle; (2) dysfunctional cognitions regarding cancer (prognosis) and cancer treatment; (3) dysfunctional fatigue-related cognitions; (4) deregulated activity pattern; (5) negative social interactions and low perceived social support. Each of these perpetuating factors corresponds to a treatment module:

**Table 2:** Instruments to assess which CBT modules are indicated

CBT Module	Instrument	Rating (RANGE)	Cut-off value
Sleep problems and deregulated sleep-wake cycle	Sleep-wake diary	Bedtimes and wake up times of 12 consecutive days and nights	Visual inspection of bedtimes and wake up times
	Sickness Impact Profile [41]: subscale Sleep and Rest	Number and type of items endorsed, weighted according to a standardised weighting scheme	Score ≥ 60
	Symptom Checklist-90 [52]: subscale Sleeping Problems	5-point Likert scale (3–15)	Score ≥ 6
Dysfunctional cognitions regarding cancer (prognosis) and cancer treatment	Impact of Event Scale [53]: subscale Intrusion subscale Avoidance	4-point Likert scale (7–28) 4-point Likert scale (8–32)	Score ≥ 10 Score ≥ 10
	Pictorial Representation of Illness and Self Measure [54]	Self-illness separation (SIS) in cm Self-fatigue separation (SFS) in cm	Fatigue-related suffering: SIS > SFS Illness-related suffering: SFS > SIS
	Illness Cognition Questionnaire [55, 56]: subscale Acceptance subscale Helplessness	4-point Likert scale (6–24) 4-point Likert scale (6–24)	Score ≤ 12 Score > 14
	Beck Depression Inventory-II Primary Care [57]	4-point Likert scale (0–21)	Score ≥ 4
	Hospital Anxiety and Depression Scale [58]: subscale Anxiety subscale Depression	4-point Likert scale (0–21) 4-point Likert scale (0–21)	Score ≥ 9 Score ≥ 9
Dysfunctional fatigue-related cognitions	Fatigue Catastrophising Scale [45]	5-point Likert scale (10–50)	Score ≥ 16
	Self-Efficacy Scale [26, 59]	4-point Likert scale (7–28)	Score ≤ 19
	Illness Management Questionnaire-factor III [60]	6-point Likert scale (9–54)	Score ≥ 30
	Anxiety for Fatigue	4-point Likert scale (8–32)	Score ≥ 14
Deregulated activity pattern	Actigraphy during 12 consecutive days	Number of days with a mean physical activity level > 66	Low-active: 0-1 Relatively-active: ≥ 2
	Sickness Impact Profile [41]: subscale Social Interactions	Number and type of items endorsed, weighted according to a standardised weighting scheme	Score ≥ 100
	Checklist Individual Strength [33]: subscale Concentration	7-point Likert scale (5–35)	Score ≥ 18
Negative social interactions and low perceived social support	Van Sonderen Social Support Inventory [61] (shortened version): subscale Negative Interactions subscale Discrepancies	4-point Likert scale (7–28) 4-point Likert scale (8–32)	Score ≥ 10 Score ≥ 14

##### Module 1: Regulation of sleep-wake cycle and improving sleep hygiene

The patient will be explained how the ‘biological clock’ can be reset, in order to establish a consistent sleep-wake pattern with regular bed and wake-up times and no day-time napping. If necessary, advice with respect to sleep hygiene will be given.

##### Module 2: Reformulate dysfunctional cognitions regarding cancer and cancer treatment

This module aims to help the patient formulate more helpful beliefs to improve his or her coping with the fact of having incurable cancer, including fear of the future, and experiencing side effects of cancer treatment. Dysfunctional beliefs will be discussed and restructured.

##### Module 3: Reformulate dysfunctional cognitions regarding fatigue

The goal is to increase self-efficacy with respect to fatigue, reduce fatigue catastrophising, and help the patient to focus less on fatigue.

##### Module 4: Regulation of activity

Two activity patterns will be distinguished on the basis of actigraphy (see *‘Outcomes’*): relatively active or low active. Some severely fatigued patients have a persistent low level of physical activity, while others have a more fluctuating activity pattern with bursts of activities followed by periods of inactivity (‘all-or-nothing behaviour’). Both activity patterns can perpetuate fatigue. Relatively active participants are helped to spread their physical, mental, and social activities more evenly over the day and week. Subsequently, participants will gradually increase their physical activity level by means of a daily walking or cycling program of their choice. The chosen activity will be gradually and systematically increased. Low active participants will be motivated to immediately start with the graded activity program. By increasing physical activity, participants’ self-efficacy with respect to physical activity and fatigue will often change positively. Eventually, participants will also increase mental and social activities.

##### Module 5: Improve social support and change unhelpful social expectations

This module is directed at modifying the patients’ unhelpful cognitions regarding their social environment, as they can maintain fatigue. Unrealistic expectations towards others are detected and disputed. Patients will practise with exercises in order to change these unhelpful cognitions and are encouraged to involve their partner in this module. Also, coping strategies in contact with others, such as family, friends, and/or colleagues, will be discussed.

After addressing the perpetuating factors of fatigue, patients will gradually work towards realising the treatment goals formulated at the start of the intervention. At the end of the intervention it is discussed how to deal with new episodes of fatigue, that may be induced when starting further lines of systemic cancer treatment.

#### Usual care and use of co-intervention

All participants will be treated for incurable cancer in concordance with national and regional cancer clinical practice guidelines of the Dutch Comprehensive Cancer Centres [36]. Participants assigned to the control group have no access to one of the two study interventions, but may be referred by their oncologist or general practitioner to physical therapists or psychologists as part of UC. Participants assigned to CBT will be asked not to follow an exercise programme as part of UC simultaneously, and participants assigned to GET will be asked not to follow a psychological intervention as part of UC simultaneously. We will collect information on whether participants have engaged in exercise programmes or psychological interventions as part of UC at all three post-randomisation assessments (T1, T2, and T3).

### Adverse events

All adverse events (AEs) and serious adverse events (SAEs) reported spontaneously by the participants or observed by the GET or CBT therapists will be recorded. All reported AEs will be followed until they have aborted, or until a stable situation has been reached. SAEs are defined as any medical occurrence that results in death, is life threatening, requires hospitalisation, results in persistent or significant disability or incapacity, or a new event of the study likely to affect the safety of participants. SAEs will be reported to the Research Ethics Committee of the University-affiliated hospital that approved the study protocol. At post-intervention assessment (T1), patients will be asked whether they think they currently experience or have experienced AEs as a result of the intervention (GET or CBT) they have received. In case of an affirmative answer, patients will be asked to specify these AEs.

### Adherence and treatment integrity

Data will be collected with respect to participants’ attendance of GET or CBT sessions, dropout from the intervention (<2 sessions attended), and therapists’ adherence to the protocol. Adherence to GET and CBT intervention protocols will be determined by means of evaluating the registration forms completed by therapists, including components of the intervention protocol that have been addressed during each session. In addition, with permission of participants, all CBT sessions will be audio taped and upon study completion a random sample of 5% will be analysed to determine treatment integrity.

### Refusal of study participation and study dropout

The researcher will record the reasons why patients do not participate, why participants dropout from the intervention, and why study assessments are not completed (T1, T2, or T3). Upon completion of the study, these reasons will be categorised, scored and analysed to gain insight into the generalisability of the findings.

### Outcomes

Outcome measures and data collection time points are listed in Table 3. The primary endpoint of this study is the post-intervention assessment (T1), 14 weeks after randomisation. Primary and secondary outcomes will be measured at baseline (T0), post-intervention (T1) and follow-up (T2, T3). Proposed mediators will only be assessed at T0 and T1.

**Table 3:** Data collection time point of all outcome measures and proposed mediators

Concept	Questionnaire	Measurement time points			
		T0	T1	T2	T3
Socio-demographics	Self-report questionnaire	X			
Medical characteristics	Medical chart review	X	X		
Primary outcome:					
Fatigue severity	CIS *fatigue severity*	X	X	X	X
Secondary outcomes:					
Fatigue	EORTC QLQ-C30 *fatigue*	X	X	X	X
Quality of life	EORTC QLQ-C30 *global health status*	X	X	X	X
Functional impairments	SIP	X	X	X	X
	EORTC QLQ-C30 *emotional functioning*	X	X	X	X
	EORTC QLQ-C30 *physical functioning*	X	X	X	X
Proposed mediators:					
Physical activity	Actigraphy during 12 consecutive days	X	X		
Physical fitness	6MWT	X	X		
Self-efficacy	SES	X	X		
Fatigue catastrophising	FCS	X	X		

*T0* baseline (pre-intervention), *T1* post-intervention/UC (14 weeks post-randomisation), *T2* first follow-up assessment (18-weeks post-randomisation), *T3* s follow-up assessment (26-weeks post-randomisation)

#### Primary outcome

Fatigue severity will be measured using the subscale *fatigue severity* (8 items, 7-point Likert scale) of the Checklist Individual Strength (CIS-fatigue) [33]. Scores range from 8–56. A score of 35 points or higher is an indication for severe fatigue. The CIS-fatigue has been used in previous intervention studies aimed at CRF and proved to be sensitive to change [23, 27]. The CIS-fatigue has good reliability (Cronbach’s alpha = 0.88) and discriminative validity [37].

#### Secondary outcomes

Fatigue will also be assessed with the symptom scale *fatigue* (3 items, 4-point Likert scale) of the European Organisation for Research and Treatment of Cancer Quality of Life Questionnaire (EORTC QLQ-C30, version 3.0). The EORTC QLQ-C30 is developed for use in clinical trials in cancer patients [38]. This instrument consists of five functional and three symptom scales in addition to a scale on global health related quality of life (HRQoL), and a number of single items assessing additional symptoms [38, 39]. Total scores on each subscale are linearly converted to a 0 to 100 scale. Higher scores represent more fatigue.

The subscale *global health status/QoL* (2 items, 7-point Likert Scale) of the EORTC QLQ-C30 will be used to measure quality of life. A high score indicates good HRQoL. The EORTC QLQ-C30 is one of the most commonly used HRQoL instruments [40] and is known to be a reliable and valid measure of the quality of life of cancer patients [38].

Functional impairments will be assessed with two instruments*.* We will include seven subscales of the Sickness Impact Profile (SIP) to assess the level of functional impairments [41]. This questionnaire measures the influence of complaints in different areas of daily functioning. The following subscales will be used: alertness behaviour, sleep, homemaking, leisure activities, mobility, social interactions, and ambulation. High scores reflect high levels of functional impairments. The SIP is known to be a reliable instrument with sufficient content validity [42]. In addition to the SIP, functional impairments will also be assessed by the subscales *emotional functioning* (4 items, 4-point Likert scale) and *physical functioning* (5 items, 4-point Likert scale, range 0 to 100) of the EORTC QLQ-C30. Raw scores for both subscales are convertible to a score of 0 to 100. A high score represents a high level of functioning.

#### Proposed mediators

Change scores (T1-T0) for each proposed mediator will be calculated and used for multiple mediation analysis. The following proposed mediators will be assessed at T0 and T1:

##### Physical activity

The level of physical activity will be assessed with actigraphy. Participants will be wearing an actometer around the ankle for twelve consecutive days and nights following T0 and T1. This actometer is a motion-sensing device based on a piezo-electric sensor recording the number of movement at five-minute intervals and with highly reproducible readings [43]. The mean daily physical activity score over twelve days can be calculated as a measure of physical activity.

##### Physical fitness

We will assess the level of physical fitness with the Six-Minute Walk Test (6-MWT). This is an easy to perform and practical submaximal exercise test that has been increasingly used across various patient populations. The 6-MWT will be conducted in an indoor corridor on a pre-measured test-course of 20 meters. Participants will be instructed to walk from one end to the other while attempting to cover as much distance as possible during the allotted time. Patients who normally use walking aids will be allowed to use them during the test. The total walking distance covered in six minutes provides an indirect measure of aerobic functional fitness [44].

##### Self-efficacy with respect to fatigue

The seven-item self-efficacy scale (SES) will be used to measure the amount of experienced control over fatigue [26]. All items are scored on a 4-point Likert scale. Higher scores are indicative for more sense of control.

##### Fatigue catastrophising

We will use the ten-item Fatigue Catastrophizing Scale to measure catastrophising in response to fatigue [45]. All items are scored on a 5-point Likert scale. Higher total scores indicate more fatigue catastrophising.

### Sample size calculation

Based on the primary outcome measure of the TIRED study, efficacy of one or both interventions is demonstrated when mean fatigue severity (CIS-fatigue) in participants assigned to GET and/or CBT is significantly lower at T1 compared to participants assigned to UC. A clinically relevant difference between the intervention arms and the UC arm of at least 6 points is expected for the primary outcome (CIS-fatigue). Per arm, a minimum number of 51 evaluable participants at T1 would be needed for a *t*-test with a power of 0.80 and a two-sided alpha of 0.025 (corrected to account for the two comparisons: GET versus UC and CBT versus UC). According to Borm et al. [46], using analysis of covariance (ANCOVA) instead of a *t*-test to analyse treatment effects on a continuous outcome measure (CIS-fatigue) increases the power and reduces the needed sample size in RCTs. This proposed ‘design factor’ for ANCOVA can be calculated by multiplying the number of participants needed for the *t*-test by 1 – *p* ^2^, where *p* is the correlation between the outcome measure at T0 and T1. Since no data on the correlation of the CIS-fatigue from earlier trials in this particular patient group were available, we used a conservative approach by assuming a weak correlation (1 – 0.10^2^ = 0.99) and thus the number of participants needed was not reduced. Anticipating an attrition rate of 30%, we aim to recruit a target sample size of 219 participants at T0 (73 participants per arm).

### Statistical analyses

The statistician who will perform data analyses will be blinded for intervention allocation. To test the efficacy of both interventions compared to UC, an ANCOVA will be performed for each intervention with *fatigue severity* (CIS-fatigue) at T1 as dependent measure, condition as fixed factor and CIS-fatigue screening score as covariate [47]. Missing data is a common problem in palliative care research and is also anticipated in our study as a result of deteriorating health or because the patient has died. Data will be primarily analysed on complete case basis, i.e. only data from evaluable participants with a T1 assessment will be used. The *p*-level is adjusted to 0.025 to account for the two primary analyses, i.e., GET versus UC and CBT versus UC. When statistically significant differences between GET versus UC and/or CBT versus UC are found, additional sensitivity analysis accounting for all randomised participants will be done to explore the impact of missing data. Several methods of imputation are available and the choice will depend on the actual circumstances of missing data. We will record the causes of missing data and careful considerations will be given to which imputation procedure should be used.

In addition, ANCOVA will be performed for the secondary outcomes (*fatigue, quality of life, and functional impairments*), with baseline score (T0) on the dependent measure as covariate. In these exploratory analyses a *p*-level of 0.05 will be used. Longer-term follow-up effects at T2 and T3 will also be tested using ANCOVA, with baseline score (T0) on the dependent measure as covariate. Again, in these explorative analyses a *p*-level of 0.05 will be used. No sensitivity analysis will be done, as the power for follow-up analyses will be limited due to the expected significant amount of attrition.

Mediation analysis will be conducted to explore the possible underlying mechanisms of the expected reduction in fatigue severity (CIS-fatigue) brought on by GET and CBT at T1. Following recommendations of Preacher and Hayes (2008) [48], we will perform multiple mediation analysis using bootstrapping to test the mediating effect of four potential mediators (i.e., changes in *physical activity, physical fitness, self-efficacy with respect to fatigue, and catastrophising in response to fatigue*). We will only perform multiple mediation analysis when there is a significant effect of one or both interventions compared to UC.

### Ethical approval

The study protocol has been reviewed and approved by the Research Ethics Committee of our University-affiliated hospital (CMO Arnhem-Nijmegen, reference no. 2012/240) and the local Ethics Committees of the participating hospitals (Hospital Gelderse Vallei, Máxima Medical Center, Isala Hospital, Canisius-Wilhelmina Hospital, Hospital Pantein, Jeroen Bosch Hospital, VieCuri Medical Center, Academic Medical Center). The study is registered in the Dutch Trial Registry (reference no. NTR3812, date registered: January 23, 2013).

## Discussion

Fatigue is one of the most prevalent symptoms compromising quality of life of incurable cancer patients receiving systemic treatment with palliative intent. Graded exercise and cognitive behavioural interventions seem promising in reducing fatigue severity based on their effectiveness in disease-free cancer patients and patients receiving cancer treatment with curative intent. To our knowledge, the TIRED study will be the first RCT determining the efficacy of GET and CBT compared to UC in reducing severe fatigue in incurable cancer patients receiving systemic treatment with palliative intent.

Recruitment of participants started in January 2013. Thus far, identifying potential study participants via nurses and oncologists for this palliative care RCT has been challenging. One common barrier for recruitment in palliative care research known from the literature is professional gatekeeping [49]. A recent systematic review by Kars et al. (2015) explored reasons for gatekeeping in palliative care research, the professionals’ perception that study participation would be too burdensome for the patients was the most reported reason [50]. Yet, we recently demonstrated that 93% of incurable cancer patients that completed a fatigue-screening questionnaire during cancer treatment with palliative intent wanted to be informed by a researcher about available interventional studies for fatigue [51]. Other important reasons for gatekeeping reported by Kars et al. (2015) included health carers’ lack of time, complicated study procedures, or study procedures that interrupt usual care processes [50]. These issues have also been observed in our study and as a result we have simplified our study procedures. For example, we originally aimed to screen for the presence of severe fatigue during a nursing consultation before the first line of systemic treatment with palliative intent began. However, nurses indicated that patients often raise several important time-consuming treatment-related questions, which hampered nurses from administering the fatigue screening. Therefore, we amended the study protocol by also allowing patients to be screened for fatigue at consultations further on during treatment. Moreover, we initially aimed to include a homogeneous sample of patients with incurable breast or colorectal cancer. Then again, poor recruitment rates during the first year, made us broaden our inclusion criterion regarding cancer type. Finally, we have extended our research collaboration with three hospitals to nine hospitals in total. All study protocol amendments have been reviewed and approved by the Research Ethics Committee of our University-affiliated hospital and the local Ethics Committees of the participating hospitals.

In conclusion, the TIRED study will provide information on the efficacy of GET and CBT compared to UC in reducing severe fatigue in incurable cancer patients, as well as on the mediators of any observed intervention effects. Other important outcome measures will include quality of life and functional impairments. If proven efficacious, one or both interventions might be offered as part of UC for this often overlooked and understudied patient group.

### Status of the trial

The TIRED-study started in January 2013 and patient recruitment is ongoing.

## Abbreviations

1RM: One-Repetition Maximum
6-MWT: Six-Minute Walking Test
CBT: Cognitive Behaviour Therapy
CIS: Checklist Individual Strength
EORTC-QLQ-C30: European Organisation for Research and Treatment of Cancer-Quality of Life Questionnaire-Core 30
FCS: Fatigue Catastrophising Scale
GET: Graded Exercise Therapy
HRR: Heart Rate Reserve
RCT: Randomised Controlled Trial
SES: Self Efficacy Scale
SIP: Sickness Impact Profile
UC: Usual Care
